# Inflammation Promotes Oxidative and Nitrosative Stress in Chronic Myelogenous Leukemia

**DOI:** 10.3390/biom12020247

**Published:** 2022-02-03

**Authors:** Dragoslava Đikić, Andrija Bogdanović, Dragana Marković, Olivera Mitrović-Ajtić, Tijana Subotički, Miloš Diklić, Milica Vukotić, Teodora Dragojević, Emilija Živković, Juan F. Santibanez, Vladan P. Čokić

**Affiliations:** 1 Department of Molecular Oncology, Institute for Medical Research, National Institute of Republic of Serbia, University of Belgrade, 11129 Belgrade, Serbia; dragana.markovic@imi.bg.ac.rs (D.M.); oliveram@imi.bg.ac.rs (O.M.-A.); tijana@imi.bg.ac.rs (T.S.); milos.diklic@imi.bg.ac.rs (M.D.); milica.tosic@imi.bg.ac.rs (M.V.); teodora.dragojevic@imi.bg.ac.rs (T.D.); ema.zivkovic55@gmail.com (E.Ž.); jfsantibanez@imi.bg.ac.rs (J.F.S.); vl@imi.bg.ac.rs (V.P.Č.); 2 Clinic for Hematology, University Clinical Center of Serbia, 11129 Belgrade, Serbia; ebogdano@eunet.rs; 3 Medical Faculty, University of Belgrade, 11129 Belgrade, Serbia; 4 Centro Integrativo de Biología y Química Aplicada (CIBQA), Universidad Bernardo O’Higgins, Santiago 8370854, Chile

**Keywords:** chronic myelogenous leukemia, inflammation, oxidative stress

## Abstract

Chronic inflammation is characterized by the production of reactive oxygen species (ROS), reactive nitrogen species, and inflammatory cytokines in myeloproliferative neoplasms (MPNs). In addition to these parameters, the aim of this study was to analyze the influence of ROS on the proliferation-related AKT/mTOR signaling pathway and the relationship with inflammatory factors in chronic myelogenous leukemia (CML). The activity of the antioxidant enzymes superoxide dismutase, glutathione peroxidase, and catalase is reduced in erythrocytes while levels of the oxidative stress markers malondialdehyde and protein carbonyl are elevated in the plasma of patients with CML. In addition, nitrogen species (nitrotyrosine, iNOS, eNOS) and inflammation markers (IL-6, NFkB, and S100 protein) were increased in granulocytes of CML while anti-inflammatory levels of IL-10 were decreased in plasma. CML granulocytes exhibited greater resistance to cytotoxic H_2_O_2_ activity compared to healthy subjects. Moreover, phosphorylation of the apoptotic p53 protein was reduced while the activity of the AKT/mTOR signaling pathway was increased, which was further enhanced by oxidative stress (H_2_O_2_) in granulocytes and erythroleukemic K562 cells. IL-6 caused oxidative stress and DNA damage that was mitigated using antioxidant or inhibition of inflammatory NFkB transcription factor in K562 cells. We demonstrated the presence of oxidative and nitrosative stress in CML, with the former mediated by AKT/mTOR signaling and stimulated by inflammation.

## 1. Introduction

Chronic myeloid leukemia (CML) is a monoclonal disease of hematopoietic stem cells (HSCs) as opposed to the polyclonal nature of normal hematopoiesis/myelopoiesis [1]. The initial molecular event is t(9;22)(q34;q11) reciprocal translocation between chromosomes that generates the *BCR-ABL* fusion oncogene [1]. Oligomerization via the coiled-coil region of BCR and deletion of the inhibitory SH3 domain of ABL results in autophosphorylation and constitutively active tyrosine kinase [2]. BCR-ABL-positive cells are characterized by genetic instability leading to CML progression from a latent chronic phase (CP) to a more advanced accelerated phase (CML-AP) and finally to an aggressive blast crisis phase (CML-BP) [1].

BCR-ABL gene fusion as a primary oncogenic event results in the activation of several signaling pathways: STAT5, Ras, MAPK, PI3K, c-jun, and c-myc [3]. These signaling pathways are important for cell metabolism, growth, proliferation, differentiation, and survival [3]. BCR-ABL kinase leads to constitutive activation of the PI3K/AKT signaling pathway by binding to the p85 PI3K regulatory subunit [4]. AKT activation is an oncogenic phenomenon that is widespread in many malignancies, and results in phosphorylation of NF-kappa B (NFkB) and mammalian target of rapamycin serine/threonine kinase (mTOR) [5]. The AKT/mTOR signaling pathway and its downstream effector p70 S6 kinase are increased in myeloproliferative neoplasm (MPN) patients, with *JAK2*V617F mutation dependance [6]. The positive effect of mTOR on cell growth refers to the initiation of mRNA translation, ribosome synthesis, expression of genes that regulate metabolism, autophagy, and cytoskeletal reorganization [7].

Small amounts of reactive oxygen species (ROS) are formed in eukaryotic cells by incomplete reduction of oxygen in physiological oxidoreduction processes during aerobic metabolism [8]. ROS includes highly reactive free radicals: superoxide anion (O_2_^−^), hydroxyl radical (OH^−^), peroxyl radical (ROO^−^), hydroperoxyl (HOO^−^), alkoxyl radical (RO), and non-radical species and oxidizing agents, which can be easily converted to radicals, such as hydrogen peroxide (H_2_O_2_), hypochlorous acid (HOCl), and singlet oxygen [9]. At lower concentrations, they act as signaling molecules in several physiological processes [9]. Higher levels of ROS lead to oxidative stress, an imbalance between prooxidants and the antioxidant defense system, which causes damage to lipids, proteins, and deoxyribonucleic acid (DNA) [9,10]. The activities of ROS are intertwined with the activities of nitrogen monoxide (NO) and other reactive nitrogen species (RNS), which are also part of physiological processes in the cell [10]. NO is formed by the synthesis of L-arginine, NADPH, and oxygen by the action of three isoforms of nitric oxide synthases (NOS) in response to mitogenic and inflammatory stimuli [11]. ROS support the activation of the AKT/mTOR signaling pathway and an enhanced iNOS frequency and nitrotyrosine levels in MPN patients [6].

In addition to having a mutagenic effect and participating in the initiation and propagation of cancer, ROS/RNS are also mediators of inflammation. We wondered whether oxidative stress alone or as part of the inflammatory process affects the activity of the mTOR signaling pathway in CML. For this purpose, we analyzed biomarkers of inflammation, oxidative, and nitrosative stress in plasma, granulocytes, and erythrocytes of CML. Using in vitro studies, we examined ROS activation of mTOR signaling and oxidative stress parameters after treatment with inflammatory cytokines.

## 2. Materials and Methods

### 2.1. Patients and Healthy Donors

This study included 30 de novo CML patients (17 men and 13 women) and 10 age-matched healthy controls (5 men and 5 women). The median age of patients was 58 years (range: 50–75) at the time of diagnosis while the median age of healthy donors was 50 years (range: 38–67). All patients signed a consent form, approved by the Local Ethical Committee, in accordance with the Declaration of Helsinki. The diagnosis of CML was based on the criteria of the World Health Organization from 2016.

### 2.2. Plasma Collection and Separation of Erythrocytes

Blood samples of CML patients and healthy controls were collected in tubes with ethylenediaminetetraacetic acid (EDTA). The plasma was separated by centrifugation and aliquots were stored at −20 °C until further analysis. Two milliliters of blood were separated and centrifuged (3000 rpm, 4 °C for 15 min) to isolate erythrocytes. The separated erythrocytes were washed three times with cold sterile physiological solution. After each centrifugation, the surface ring of white blood cells was removed. Erythrocytes were resuspended in saline (1:1) and the aliquots were stored at −70 °C until enzyme analysis.

### 2.3. Biochemical Assays

The total antioxidant capacity and quantity of oxidative products of lipids and proteins were measured in plasma. The ferric-reducing ability/antioxidant power (FRAP) assay is a redox-linked colorimetric method, which is based on the reduction of the yellow ferric-tripyridyltriazine complex (Fe (III)-TPTZ) to a blue-colored ferro complex (Fe (II)-TPTZ), at low pH [12]. Lipid peroxidation was estimated by a spectrophotometric assay based on the absorption maximum of the malondialdehyde (MDA) complex with thiobarbituric acid (TBA) in an acidic environment and at high temperatures [13]. Determination of the protein carbonyl (PC) concentration in oxidatively modified plasma proteins was based on the method of Levin and associates [14]. The amount of protein carbonyl was expressed in relation to the concentration of total proteins in the sample. The activity of antioxidant enzymes was measured in the lysates of erythrocytes. The method used for the determination of superoxide dismutase (SOD) activity was based on the superoxide-anion-dependent autoxidation of adrenaline in an alkaline environment [15]. SOD removes the superoxide anion radical and enzyme activity was proportional to the inhibition of autooxidation monitored spectrophotometrically. Biochemical analysis of catalase (CAT) activity was performed using the method of Aebi based on the decomposition rate of hydrogen peroxide (H_2_O_2_) in the presence of the enzyme [16]. To determine glutathione peroxidase (GPx) activity, an indirect method was used to monitor the decrease in the absorption of NADPH at 340 nm in the presence of glutathione reductase (GR). The spectrophotometric method used to investigate GR activity was based on the monitoring of NADPH oxidation in the reduction reaction of oxidized glutathione [17]. The hemoglobin concentration in lysate erythrocytes was determined spectrophotometrically after the freezing/thawing method by treatment with Drapkin’s solution (potassium ferricyanide (K_3_Fe(CN)_6_) and potassium cyanide (KCN)). The activity of antioxidant enzymes in erythrocytes is shown in relation to the hemoglobin concentration (U/mg Hb).

### 2.4. ELISA Assay

Plasma samples obtained from 18 patients and 4 healthy controls were used to assess IL-6 levels using an ELISA kit (R&D Systems, Minneapolis, MN, USA), according to the manufacturer’s instructions. All samples were tested in duplicate, and data were obtained by a standard curve that was created using the recombinant standards and expressed as the average of IL-6 levels in pg/mL for each group. Measurements were performed on an ELISA Multiscan Plus plate reader (Labsystems, Vantaa, Finland).

### 2.5. Immunocytochemistry

The granulocytes of patients and healthy subjects were isolated after separation of cell fractions by Lymphocyte Separation Medium (LSM, Capricorn Scientific GmbH, Ebsdorfergrund, Germany) and hypotonic lysis of precipitated erythrocytes. Granulocytes were resuspended in the medium (0.4 × 10^6^ cell/mL) and 500 µL of the cell suspension were used to prepare the cytospin slide by centrifugation (1000 rpm, RT, for 5 min). Slides were fixed in acetone and stored at −20 °C until use. Before proceeding with immunocytochemistry, the microscopic slides were washed with phosphate buffer solution (PBS). Endogenous peroxidase was blocked with 3% H_2_O_2_ solution for 10 min. The slides were incubated with primary antibody pAKT, NOS2, nitrotyrosine (R&D Systems, Minneapolis, MN, USA), pmTOR, p-pS6K, NOS3 and NFkB p65 (Santa Cruz Biotechnology, Dallas, TX, USA), S100 and p53 (Dako, Glostrup, Denmark), p-p53 (phospho S15) (Abcam, Cambrige, UK), and IL-6 (Novocastra, Leica Biosystems, Newcastle, UK) at +4 °C, overnight. After incubation with biotinized anti-rabbit immunoglobulins, the cells were treated with a streptavidin conjugated with horseradish peroxidase (UltraVision Detection System, HRP, Thermo Scientific, UK). Finally, the slides were incubated in a solution of substrate-chromogen (Liquid DAB+Substrate Chromogen System, Dako, Glostrup, Denmark). Mayer’s hematoxylin was used for the contrast. The count was performed on 700 or 800 cells in each cytospin. The slides were analyzed using an Olympuse Provis AX70 microscope, Tokyo, Japan.

### 2.6. Tetrazolium Salt Reduction Test, MTT

The colorimetric assay used to assess the cell viability was based on the reduction of tetrazolium salt MTT (3-4,5-dimethylthiazole-2,5-diphenyltetrzolium bromide) by mitochondrial dehydrogenases to form an insoluble purple formazan. This test is suitable for measuring the number of viable cells, their activity, and proliferation [18]. We used H_2_O_2_-treated and -untreated granulocytes in the test. The granulocytes of patients and controls were isolated after separation of cell fractions by Lymphocyte Separation Medium (LSM, Capricorn Scientific GmbH, Ebsdorfergrund, Germany) and hypotonic lysis of precipitated erythrocytes. The cells were resuspended in an RPMI-1640 medium (Capricorn Scientific GmbH, Ebsdorfergrund, Germany) supplemented with 10% fetal calf serum (FCS) (SigmaAldrich, St. Louis, MO, USA), L-glutamine, and penicillin/streptomycin (Capricorn Scientific GmbH, Ebsdorfergrund, Germany) and incubated at 37 °C/5% CO_2_ before MTT (5 mg/mL) was added. The cultures were incubated for 3–4 h at 37 °C. The reduction process was stopped by adding 10% SDS acidified with 1 N HCl. Dissolution of the resulting blue dye was continued overnight in an incubator. The absorbance was read at 540 nm on an ELISA reader (RT-6100, Rayto, Shenzhen, China). The ratio of the absorbances of treated to untreated living cells was used to measure the viability.

### 2.7. In Vitro Study

To examine whether ROS can alter the activity of AKT/mTOR signaling, we treated CML and control granulocytes and K562 cells, an erythroid/megakaryocytic precursor cell line derived from chronic myeloid leukemia patients in a blast crisis (American Type Culture Collection, Gainthesburg, MD, USA), with 0.5 mM and 1 mM H_2_O_2_. The cells were resuspended in an RPMI-1640 medium (Capricorn Scientific GmbH, Ebsdorfergrund, Germany) supplemented with 10% fetal calf serum (FCS) (Sigma-Aldrich, St. Louis, MO, USA), L-glutamine, and penicillin/streptomycin (Capricorn Scientific GmbH, Ebsdorfergrund, Germany) and incubated for 2 h at 37 °C/5% CO_2_ before performing the experiments. Two groups were pretreated with antioxidant N-acetylcysteine (NAC, Sigma-Aldrich St. Louis, MO, USA) at a final concentration of 3 mM, 30 min before adding the H_2_O_2_. One group was treated with 3 mM NAC alone. Untreated group was used as control. After the treatment, granulocyte suspensions were used for the preparation of cytospins and K562 cells were used for protein isolation and Western blotting.

The second experiment was designed to examine the link between oxidative stress and inflammation. First, 1 group of K562 cells was treated with interleukin 6 (IL-6, 20 ng/mL) and then 2 groups were pre-treated with 15 µM of NFkB inhibitor (JSH23, Merck, Darmstadt, Germany) or NAC (3 mM) before adding IL-6 (20 ng/mL). Treatments with H_2_O_2_ (1 mM) and/or NAC (3 mM) were used as a positive and negative control. K562 cells were used for the preparation of cytospins incubated with primary antibody 8-hydroxyguanine (8-OhdG, Santa Cruz Biotechnology, Dallas, TX, USA). The MDA was measured in K562 cell lysates and cell culture medium.

### 2.8. Protein Isolation and Immunoblotting

For protein isolation, cells were lysed in chilled RIPA buffer (50 mM Tris-HCl, pH 7.6, 150 mM sodium chloride, 1% Triton x-100, 1% sodium deoxycholate, 0.1% sodium dodecyl sulphate, 2 mM EDTA, 1 mM DTT, 50 mM sodium fluoride) with inhibitor cocktail (Thermo Fisher Scientific, Rockford, IL, USA) and sodium-orthovanadate (1 mM). After centrifugation and supernatant collection, the protein aliquots were stored at −70 °C until analysis. For Western blotting, equal amounts of protein samples were run on polyacrylamide gels under reducing conditions and transferred to PVDF transfer membranes (Thermo Scientific, Rockford, IL, USA). Membranes were probed with primary antibodies to AKT and pAKT (S473) (R&D Systems, Minneapolis, MN, USA), or pmTOR (Ser2248) and mTOR (Cell Signaling Technology, Danvers, MA, USA). Β-actin (R&D Systems, Minneapolis, MN, USA) was used as loading control after the membrane was cut using a molecular weight marker. Peroxidase-conjugated goat antirabbit immunoglobulin (Santa Cruz Biotechnology, Dallas, TX, USA) and goat anti-mouse immunoglobulin (Thermo Scientific, Rockford, IL, USA) were used as secondary antibodies. The AKT, pAKT, mTOR, pmTOR, and β-actin protein levels were imaged with a ChemiDoc Imaging System (Bio-Rad Laboratories, Hercules, CA, USA) and estimated by densitometric scanning of the blots using the Image Lab (Bio-Rad Laboratories, Inc. Version 6.0.0.25, Hercules, CA, USA) software tool and normalized to β-actin.

### 2.9. Statistical Analysis

One-way ANOVA and Dunnett’s post test were applied using Prism 4 software (GraphPad Software Inc., San Diego, CA, USA). The results are expressed as the mean ± SEM, and differences at *p* < 0.05 were accepted as the level of significance.

## 3. Results

### 3.1. Oxidative Stress in Chronic Myeloid Leukemia

**Figure 1:**
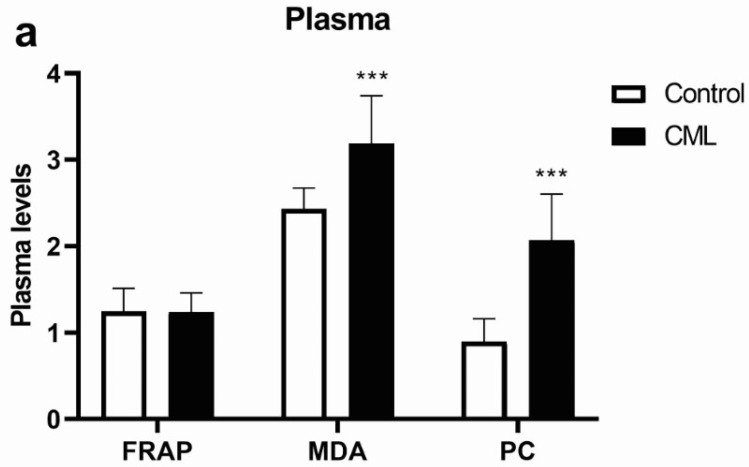

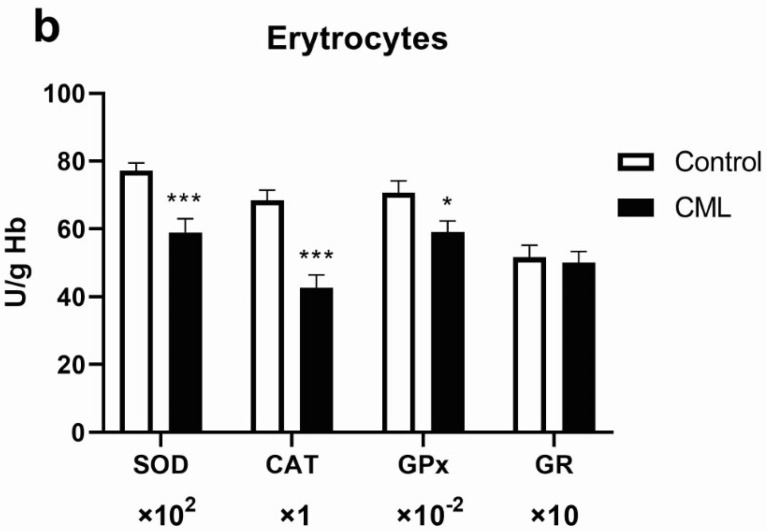
Parameters of oxidative stress and antioxidants in the blood of patients with chronic myeloid leukemia (CML). (**a**) Ferric-reducing antioxidant power (FRAP, µmol Fe/mL), malondialdehyde (MDA, nmol/mL), and protein carbonyl (PC, nmol/mg protein) levels in the plasma of patients with CML (*n* = 17) and healthy subjects (Control, *n* = 8). (**b**) Activity of superoxide dismutase (SOD), catalase (CAT), glutathione peroxidase (GPx), and glutathione reductase (GR) in erytrocytes of patients with CML (*n* = 16) and healthy subjects (Control, *n* = 10). Values are mean ± SEM. * *p* < 0.05 and *** *p* < 0.001 vs. control.

Using different colorimetric methods, we determined the levels of MDA and PC, biomarkers of oxidative stress in plasma. The levels of MDA and PC were significantly higher in the plasma of patients with CML compared to healthy subjects (*p* < 0.001, Figure 1a). The total antioxidant capacity of plasma (FRAP) determined as the reducing power of plasma in patients with CML did not differ significantly from the values of healthy controls. In contrast, a significant reduction in the antioxidant protection capacity was found in the erythrocytes of patients with CML compared to healthy subjects (Figure 1b). After measuring the activity of antioxidant enzymes in the lysate of erythrocytes, we observed a decrease in SOD activity (*p* < 0.001, Figure 1b). CAT activity was decreased by 37.6% (*p* < 0.001, Figure 1b) and GPx activity by 16.4% (*p* < 0.05, Figure 1b) in CML erythrocytes compared to controls. Examining the activity of GR in patients with CML, we did not find a difference in relation to the healthy subjects. The biochemical analysis showed that the blood markers of oxidative stress were increased in CML while antioxidant enzymes activities were mostly reduced.

### 3.2. Nitrosative Stress and Inflammation in Chronic Myeloid Leukemia

**Figure 2:**
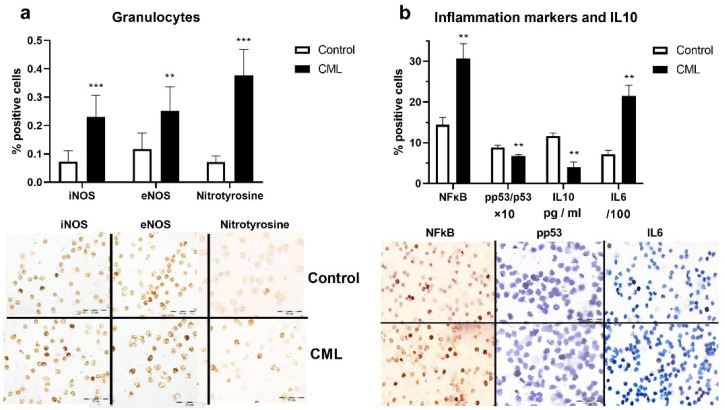
Nitric oxide synthases, nitrotyrosine, and inflammation marker expression in granulocytes and IL10 in the plasma of patients with chronic myeloid leukemia (CML). (**a**) Inducible nitric oxide synthase (iNOS), endothelial NOS (eNOS), and nitrotyrosine levels in the granulocytes of patients with CML (*n* = 13) and healthy subjects (Control, *n* = 6). In total, 700 cells per cytospin were counted. (**b**) Frequency of NFkB, IL-6, and active/total p53 ratio in granulocytes and the IL10 level in the plasma of patients with CML (*n* = 13) and healthy subjects (Control, *n* = 6). In total, 800 cells per cytospin were counted using a computer supported imaging system (Analysis Pro 3.1) connected to a light microscope with an objective magnification of 40. Below the graphs, sections of cytospins from patients and healthy controls after immunocytochemical staining are shown (40× objective magnification). Values are mean ± SEM. ** *p* < 0.01; and *** *p* < 0.001 vs. control.

Considering the connection between oxidative and nitrosative stress, we examined the expression of iNOS and eNOS in the granulocytes of patients with CML and the corresponding controls. The percentage of iNOS- (*p* < 0.001, Figure 2a) and eNOS-positive (*p* < 0.01, Figure 2a) cells were significantly higher in patients with CML compared to healthy subjects. The expression level of nitrothyrosine, a marker of nitrosative stress, was increased in the granulocytes of patients with CML compared to controls (*p* < 0.001, Figure 2a). The markers of nitrosative stress were increased in the blood of patients with CML. Since oxidative stress and granulocytes are part of the inflammatory response [19], we evaluated the markers of inflammation in granulocytes. CML patients showed a statistically higher quantity of proinflammatory NFkB compared to controls (*p* <0.01, Figure 2b). It has been reported that constitutive activation of NFκB reduces the tumor suppressor activity of p53 [20]. In the present study, the frequency of activated tumor suppressor p53 protein was significantly reduced in patients with CML (*p* < 0.01, Figure 2b). We detected a significantly higher frequency of proinflammatory IL-6-positive granulocytes in patients with CML compared to controls (*p* < 0.01, Figure 2b). As IL10 is known to have antioxidant and anti-inflammatory effects [21], we determined its concentration in plasma. A significantly lower concentration of this anti-inflammatory cytokine was found in patients with CML than in healthy subjects (*p* < 0.01, Figure 2b). The presented pro- and anti-inflammatory balance is shifted toward inflammation in CML.

### 3.3. Sensitivity of Granulocytes to Oxidative Stress and Induction of the Akt/mTOR Signaling Pathway

**Figure 3:**
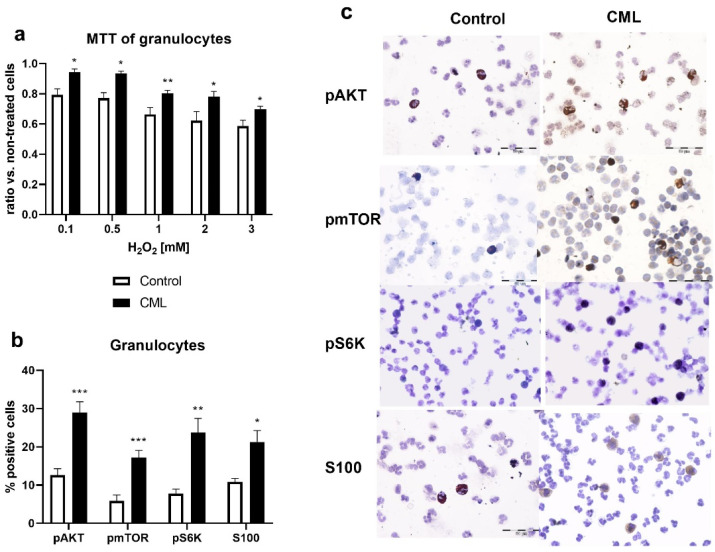
MTT test and AKT/mTOR signaling pathway in the granulocytes of patients with chronic myeloid leukemia (CML). (**a**) MTT test after treatment of granulocytes of patients with CML (*n* = 8) and healthy subjects (Control, *n* = 6) with presented concentrations of H_2_O_2_. (**b**) Percentage of pAKT, pmTOR, pS6K, and S100 protein-positive granulocytes of patients with CML (*n*= 17) and healthy subjects (Control, *n* = 6). In total, 800 cells per cytospin were counted using a computer supported imaging system (Analysis Pro 3.1) connected to a light microscope with an objective magnification of 40. (**c**) Granulocytes of patients and healthy controls after immunocytochemical staining with anti-pAKT, anti-pmTOR, anti-pS6K, and anti-S100 antibodies (40× objective magnification) corresponding to graph b). Values are mean ± SEM. * *p* < 0.05; ** *p* < 0.01; and *** *p* < 0.001 vs. control.

We used the MTT test to examine the sensitivity of granulocytes to different concentrations of H_2_O_2_ as an inducer of oxidative stress (Figure 3a). The general results showed greater viability of granulocytes originating from CML patients compared to controls (Figure 3a). In both groups, the number of living cells decreased with an increasing H_2_O_2_ concentration, but the granulocytes of the patients with CML showed greater resistance to the oxidative effects of H_2_O_2_. The frequency of myeloid-related S100 protein, participating in inflammation, was significantly increased in patients with CML compared to healthy controls (*p* < 0.05, Figure 3b). It has been reported that the interaction between the NFκB and Akt/mTOR signaling pathways promotes inflammation [22]. Using immunocytochemistry, we determined the activity of Akt/mTOR signaling in granulocytes. The percentages of the phosphorylated form of AKT, mTOR, and S6K kinases were increased in CML compared to the controls (*p* < 0.01, *p* < 0.001, Figure 3b). According to the presented results, inflammation signaling and factors are increased in CML.

### 3.4. Induction of the mTOR Signaling Pathway by Oxidative Stress in Granulocytes

**Figure 4:**
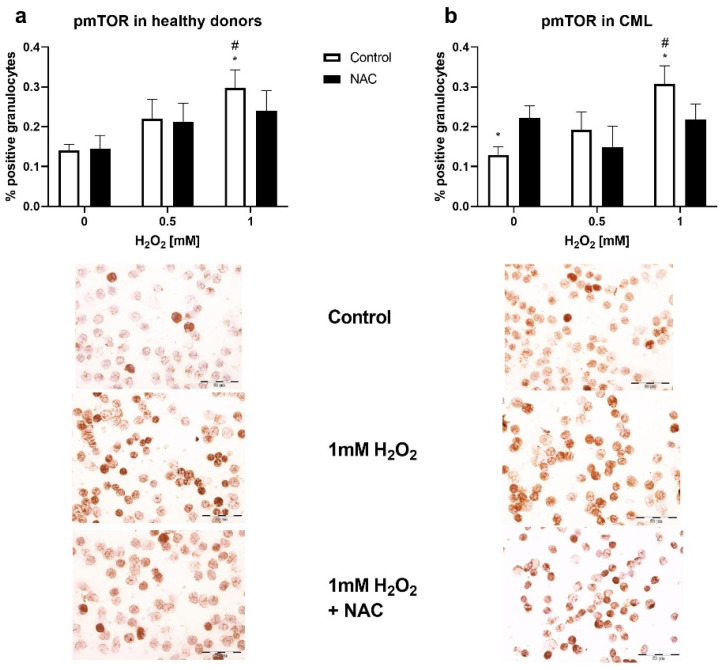
Oxidative stress induction of mTOR signaling in the granulocytes of patients with chronic myeloid leukemia (CML). (**a**) pmTOR expression in the granulocytes of healthy donors (*n* = 5) after treatment with different doses of H_2_O_2_ (0.5 mM and 1 mM) and/or antioxidant N-acetyl-cysteine (NAC, 3 mM); (**b**) pmTOR levels in the granulocytes of patients with CML (*n* = 6) treated with different doses of H_2_O_2_ (0.5 mM and 1 mM) and/or NAC (3 mM). In total, 800 cells per cytospin were counted using a computer supported imaging system (Analysis Pro 3.1) connected to a light microscope with an objective magnification of 40. The images below the graphs represent the expression of pmTOR in untreated and treated granulocytes (40× objective magnification). Values are mean ± SEM. * *p* < 0.05 vs. NAC; ^#^ *p* < 0.05 vs. Control (0) non-treated cells.

The granulocytes of patients and healthy individuals were treated with H_2_O_2_ and antioxidant (NAC) to examine the effect of oxidative stress on the activity of mTOR the signaling pathway. The immunocytochemistry showed a dose-dependent increase in phosphorylated mTOR expression in both experiments. The significant increase in kinase activity was observed after treatment with 1 mM H_2_O_2_ compared to untreated cells (*p* < 0.05, Figure 4a,b). The pre-treatment with NAC reduced the stimulation by H_2_O_2_. In the granulocytes of patients with CML, treatment with NAC induced phosphorylation of mTOR, indicating redox sensitivity of the kinase (*p* < 0.05, Figure 4b).

### 3.5. Induction of the Akt/mTOR Signaling Pathway by Oxidative Stress in K562 Cells

**Figure 5:**
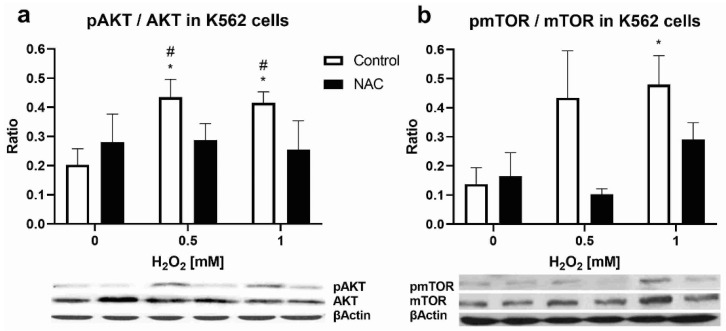
Oxidative stress induction of AKT/mTOR signaling in K562 cells: (**a**) pAKT/AKT ratio in K562 cells after treatment with different doses of H_2_O_2_ (0.5 mM and 1 mM) and/or antioxidant N-acetyl-cysteine (NAC, 3 mM); (**b**) pmTOR/mTOR ratio in K562 cells after treatment with different doses of H_2_O_2_ (0.5 mM and 1 mM) and/or NAC (3 mM). Values are mean ± SEM (*n* = 4–5). * *p* < 0.05 vs. non-treated cells; ^#^ *p* < 0.05 vs. NAC.

In the experiment, erythroleukemic K562 cells were treated with H_2_O_2_ (0.5mM, 1 mM) and NAC (3 mM) for 30 min. Immunoblot of the isolated proteins determined the expression of pAKT and AKT and pmTOR and mTOR. After the application of H_2_O_2_, the ratio of active and total forms of AKT was increased concerning the control (*p* < 0.05, Figure 5a). The increase in mTOR activity was statistically significant only after treatment with a higher dose of H_2_O_2_ (1 mM, *p* < 0.05, Figure 5b). Pre-treatment with NAC reversed the stimulatory effect of H_2_O_2_ (Figure 5a,b).

### 3.6. Markers of Oxidative Stress in K562 Cell Culture after Treatment with Hydrogen Peroxide and Interleukin 6

**Figure 6:**
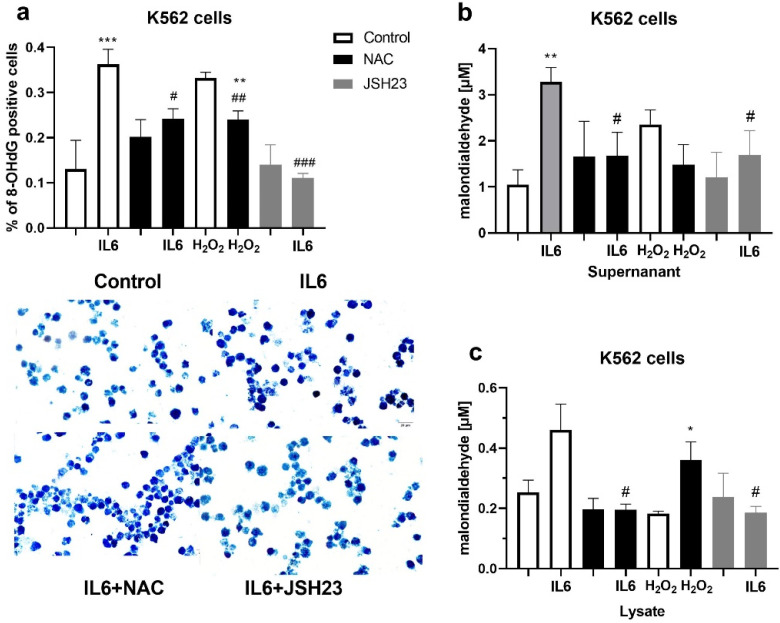
Oxidative stress markers in K562 cell and culture medium: (**a**) Expression of 8-hydroxyguanine (8-OHdG) in K562 cells after treatment with interleukine 6 (IL-6, 20 ng/mL), H_2_O_2_ (1 mM), and/or the inhibitors N-acetyl-cysteine (NAC, 3 mM) and JSH23 (15 µM). In total, 700 cells per cytospin were counted using a computer supported imaging system (Analysis Pro 3.1) connected to a light microscope with an objective magnification of 40. The images below the graphs represent the expression of 8-OHdG in cytospins of untreated and treated K562 cells (40× objective magnification). (**b**) Malondialdehyde (MDA) levels in cell culture medium after treatment with IL-6 (20 ng/mL), H_2_O_2_ (1 mM), and/or the inhibitors NAC (3 mM) and JSH23 (15 µM); (**c**) MDA levels in K562 cell lysates after treatment with IL-6 (20 ng/mL), H_2_O_2_ (1 mM), and/or the inhibitors NAC (3 mM) and JSH23 (15 µM). Values are mean ± SEM * *p* < 0.05, ** *p* < 0.01, and *** *p* < 0.001 vs. non-treated cells. ^#^ *p* < 0.05, ^##^ *p* < 0.01, and ^###^ *p* < 0.001 vs. IL-6 only.

In order to examine the influence of inflammation on oxidative stress, erythroleukemic K652 cells were treated with IL-6 and inhibitor of NFkB (JSH23). Treatments with H_2_O_2_ (1 mM) and NAC (3 mM) were used as a positive and negative control, respectively. 8-hydroxyguanine (8-OHdG) is an indicator of oxidative DNA damage. The immunocytochemical expression of 8-OHdG was significantly higher in K562 cells treated with IL-6 (*p* < 0.001, Figure 6a) and H_2_O_2_ (*p* < 0.01, Figure 6a) compared to untreated cells. The pre-treatment with NAC significantly reduced the percentage of positive cells after treatment with IL-6 (*p* < 0.05, Figure 6a) and H_2_O_2_ (*p* < 0.01, Figure 6a). The inhibitor JSH23 reversed the effect of IL-6 (*p* < 0.001, Figure 6a) on 8-OHdG expression in K562 cells. We examined the MDA quantity in lysates of K562 cells and culture medium. Treatment with IL-6 increased the concentration of MDA in the supernatant obtained after centrifugation of K562 cells (*p* < 0.01, Figure 6b). The pre-treatment with NAC and JSH23 significantly reduced augmentation of MDA in medium (*p* < 0.05, Figure 6b) and in lysates of K562 cells (*p* < 0.05, Figure 6c).

## 4. Discussion

Numerous studies have shown increased ROS production and the existence of oxidative stress in MPN [23,24,25]. Neutrophils are the main source of ROS in the blood, which is especially pronounced in MPN. The balance between oxidants and antioxidants is disturbed in CML in the presented study. In the plasma of patients with CML, the concentrations of lipid peroxidation products and carbonyl proteins, formed by protein oxidation, were increased. Our results are consistent with the results of studies that showed increased levels of MDA and carbonyl proteins in both the chronic and exacerbation phases of CML [26,27]. The high MDA concentration is associated with a decrease in non-enzymatic antioxidant protection activity and increases with disease progression [28].

The antioxidant capacity in patients with CML investigated in this study refers to the activity of antioxidant protection enzymes in erythrocyte lysates. A decrease in SOD, CAT, and GPx activity was measured in patients with CML. The results are consistent with our previous research in Ph-MPN with a significant difference between MPN subtypes [6]. We also detected aberrant expression of oxidative stress-induced genes in circulatory CD34^+^ cells [6]. The long-term activation of STAT5 by BCR/ABL kinase could be responsible for decreased intracellular expression of CAT and glutaredoxin 1 [29]. Similarly, the other study suggests the existence of a feed-forward loop in which BCR-ABL1 enhances its own mutation rate in an STAT5-ROS-dependent manner [30].

The high levels of oxidative macromolecules indicate the degree of oxidation-reduction imbalance in the circulation. The biological significance of the observed condition depends on the cell type and antioxidant capacity [6]. In our previous work, increased CAT activity was observed in primary myelofibrosis, as the final stage of MPN development [6]. Moreover, the granulocytes of CML patients showed greater resistance to the cytotoxic action of H_2_O_2_ compared to healthy subjects, indicating better adaptation to high ROS concentrations. This represents an advantage in survival under the altered microenvironmental conditions that exist in CML.

NO plays a dual role in tumor biology because it has both a stimulatory and preventive effect on disease development by altering the activity of regulatory molecules [11]. The classical pathway of NO enzyme synthesis, L-arginine–NOS, is the main source of NO in the body, and iNOS is a key isoform of this enzyme in NO signaling associated with inflammation and carcinogenesis [19,31]. Elevated levels of NOS were observed in CML patients compared to controls. Our previous study also showed a positive correlation between nitrite/nitrate plasma concentrations and iNOS expression in patients’ granulocytes, indicating that elevated NO in these malignancies is probably caused by iNOS activity [6]. Nitrothyrosine is one of the stable end products of NO metabolism and a marker of cumulative activity of iNOS [32]. Nitrothyrosine is most often formed after the reaction of peroxynitrite with proteins that contain tyrosine amino acid residues. Protein nitration affects one of the most important mechanisms of cellular regulation, the cyclic change between phosphorylated and unphosphorylated forms of tyrosine in proteins [33]. We demonstrated a positive correlation between the expression of iNOS and nitrotyrosine in granulocytes, indicating that iNOS is important for the onset of this modification [6].

iNOS activity in cancer has both a positive and negative impact on disease progression [31]. NO enhances the oncogenic properties of p53 or activates HIF-1α, Akt, and ERK signaling pathways, which further leads to increased proliferation, activation of antiapoptotic pathways, and metastasis formation [34]. The p53 protein is a homotetrameric transcription factor that participates in the removal of damaged DNA by reparation or apoptosis. Its inactivation or reduced expression are characteristics of many cancers and may be due to oxidative stress [35]. Phosphorylation is important for p53 stabilization and activation in the physiological context and in the earliest stages of tumor development [36]. In our study, patients with CML had significantly lower p53 protein activity in their granulocytes. This data, combined with the presented oxidative stress, confirms that the primary tumor-suppressive function of p53 in cancers is to decrease ROS by regulating antioxidant and metabolism genes rather than apoptosis and cell-cycle arrest [34,35]. According to Velu et al. [37], reduced p53 expression could be a consequence of oxidative stress when under the influence of ROS and RNS, resulting in S-glutathionylation of the protein DNA-binding domain. Decreased p53 activity under oxidative stress conditions contributes to genomic instability critical for cancer development.

In 2013, Hasselbalch hypothesized that constant inflammation could cause damage to stem cells in the bone marrow because it leads to oxidative DNA damage and creates a microenvironment conducive to the induction of mutations [38]. IL-6 is a key cytokine whose secretion by myeloid cells mostly affects the pathogenesis of CML. BCR/ABL activity controls Il-6 expression, thereby establishing a paracrine feedback loop that sustains CML development [39]. Elevated serum IL-6 levels, released by monocytes, macrophages, and T cells, have been reported in leukemia patients [40]. We have shown that treatment with IL-6 causes oxidative changes in cellular macromolecules, DNA, and lipids. The nature of these changes and the fact that the antioxidant mitigates the harmful effects of cytokine indicate that ROS are potential mediators of action. Another component of the complex action of IL-6 is the NFkB transcription factor, which regulates immune processes and tumor-promoting inflammation [41]. Blockade of NFkB transcriptional activity reduced oxidative damage in vitro. Cross-signaling between NFkB and IL-6 has been shown in vascular inflammation and glioblastoma [42,43]. The promoter region of the IL-6 gene has an NFkB-binding site [44]. Since the function of IL-6 is recruitment of neutrophils, we showed that the expression of this cytokine was significantly increased in the peripheral blood granulocytes of patients with CML associated with higher levels of NFkB. NFκB is an inducible transcription factor, sequestered in the cytoplasm by a family of inhibitory proteins. Degradation of inhibitors is followed by nuclear translocation and NFkB factor activation [41]. Higher amounts of NFkB in CML granulocytes create a condition for a more intense immune response after inflammatory stimulation compared to controls.

As part of the proinflammatory milieu in CML, we detected a decrease in IL10 values. High s100 expression has been detected in acute myeloid leukemia, which corresponds to the high values of this protein in the examined granulocytes of patients with CML [45].

Aberrant mTOR activation has been observed in hematologic malignancies, including MPN [46], indicating that mTOR regulation is crucial for normal hematopoiesis [47]. In our previous work, activation of the mTOR pathway was most prominent in the bone marrow of primary myelofibrosis (PMF) and granulocytes of PMF and essential thrombocythemia patients [6]. The activity of the mTOR signaling pathway was confirmed by high levels of pmTOR and its downstream effector, S6 kinase, in the examined granulocytes of patients with CML. This is consistent with the results of other researchers, who have shown constitutive activation of mTOR and its effectors S6K and 4E-BP1 in the presence of BCR/ABL kinase [48].

High concentrations of H_2_O_2_ were observed in the neutrophils of CML patients both before and after antitumor therapy [49]. There are contradictory data showing that mTOR can be both activated and inhibited by oxidative stress, depending on the cell type in which it is expressed and the type of oxidant [50,51]. In our experiments, we showed that H_2_O_2_ has a stimulatory effect on mTOR signaling pathway activity, both in patients and healthy controls. The balance between kinase and phosphatase activity determines the signal intensity of the Akt/mTOR pathway [52]. Mutation or loss of function of PTEN phosphatase, whose activity is also regulated by the redox potential, is known to be associated with increased mTOR activity [53]. Our results indicated redox sensitivity of the mTOR complex in CML since treatment with both oxidant and antioxidant increased its activity compared to untreated granulocytes.

Since granulocytes are very sensitive and reactive cells whose viability in culture decreases rapidly, part of the experiment was done with K562 cells that exhibit constitutive activation of BCR/ABL tyrosine kinase. The results of these experiments confirmed the stimulatory effect of H_2_O_2_ on the mTOR signaling pathway through increased AKT and mTOR phosphorylation. We reported earlier that treatment of Hel cells with H_2_O_2_ induces AKT/mTOR pathway activity [6]. The influence of ROS on the activity of signaling pathways should be viewed as part of the redox state change as an important factor in the regulation of intracellular processes [54]. It is assumed that TOR in the cell is in a reduced and oxidized form, and their relationship is determined by factors that otherwise regulate the cell’s redox potential, including ROS [50,55]. Tumor cells may be more resistant to oxidative stress than normal cells because their high level of ROS production is often accompanied by increased antioxidant protection capacity. In this way, they gain a proliferative advantage over healthy cells because they avoid the harmful effects of ROS, and at the same time, as a source of reactive species, induce apoptosis of surrounding healthy cells [9,56]. In our experiment, the patient’s granulocytes showed greater resistance to the harmful effects of H_2_O_2_, probably because of better adaptation to high concentrations of ROS [57].

## 5. Conclusions

In our study, we showed an increase in markers of oxidative and nitrosative modifications in CML while the activity of most antioxidant enzymes was significantly reduced. Under conditions of oxidative stress, increased activation of AKT and mTOR kinases was detected in BCR/ABL-positive cells. We support the thesis that high levels of ROS activate proximal signaling pathways that promote proliferation and survival of cancer cells. This may be due to the better adaptation of altered cells that retain vitality despite damage. Our results revealed that inflammation stimulated oxidation via NFκB factor. The oxidative status in CML patients may indicate new diagnostic and prognostic markers that can be detected in peripheral blood granulocytes and provide new possibilities for therapy improvement.
